# (Pro)renin receptor signaling in hypothalamic tyrosine hydroxylase neurons is required for obesity-associated glucose metabolic impairment

**DOI:** 10.1172/jci.insight.174294

**Published:** 2024-02-13

**Authors:** Shiyue Pan, Lucas A.C. Souza, Caleb J. Worker, Miriam E. Reyes Mendez, Ariana Julia B. Gayban, Silvana G. Cooper, Alfredo Sanchez Solano, Richard N. Bergman, Darko Stefanovski, Gregory J. Morton, Michael W. Schwartz, Yumei Feng Earley

**Affiliations:** 1 Departments of Pharmacology and Physiology & Cell Biology and; 2 Center for Molecular and Cellular Signaling in the Cardiovascular System, University of Nevada, Reno, Reno, Nevada, USA.; 3 Diabetes and Obesity Research Institute, Cedars-Sinai Medical Center, Los Angeles, California, USA.; 4 New Bolton Center, School of Veterinary Medicine, University of Pennsylvania Philadelphia, Pennsylvania, USA.; 5 University of Washington Medicine Diabetes Institute, University of Washington, Seattle, Washington, USA.

**Keywords:** Endocrinology, Metabolism, Diabetes, Glucose metabolism

## Abstract

Glucose homeostasis is achieved via complex interactions between the endocrine pancreas and other peripheral tissues and glucoregulatory neurocircuits in the brain that remain incompletely defined. Within the brain, neurons in the hypothalamus appear to play a particularly important role. Consistent with this notion, we report evidence that (pro)renin receptor (PRR) signaling within a subset of tyrosine hydroxylase (TH) neurons located in the hypothalamic paraventricular nucleus (PVN^TH^ neurons) is a physiological determinant of the defended blood glucose level. Specifically, we demonstrate that PRR deletion from PVN^TH^ neurons restores normal glucose homeostasis in mice with diet-induced obesity (DIO). Conversely, chemogenetic inhibition of PVN^TH^ neurons mimics the deleterious effect of DIO on glucose. Combined with our finding that PRR activation inhibits PVN^TH^ neurons, these findings suggest that, in mice, (a) PVN^TH^ neurons play a physiological role in glucose homeostasis, (b) PRR activation impairs glucose homeostasis by inhibiting these neurons, and (c) this mechanism plays a causal role in obesity-associated metabolic impairment.

## Abstract 2

The activation of (pro)renin receptor signaling in tyrosine hydroxylase neurons in the hypothalamic paraventricular nucleus disrupts glucose regulation and plays a pivotal role in the metabolic dysfunction associated with obesity.

## Graphical abstract

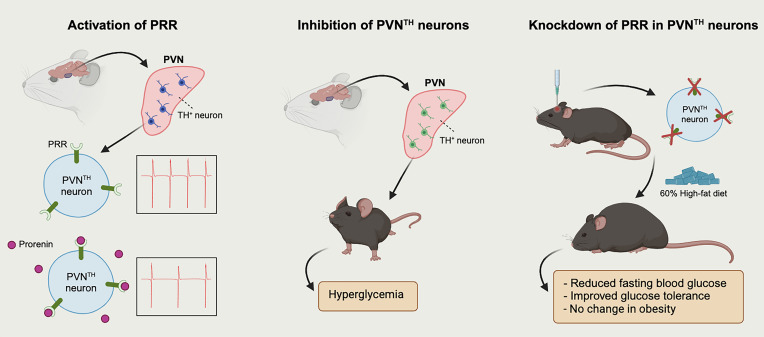

## Introduction

The term “glucose homeostasis” refers to a complex set of coordinated responses that collectively maintain the circulating glucose level within narrow limits by balancing glucose entry into and removal from the bloodstream over time. While the pancreas and liver are vital to this process, growing evidence implicates a key role for the CNS, with the hypothalamus playing a particularly important role (1–3). Experimental activation of a subset of neurons in the hypothalamic ventromedial nucleus (VMN), for example, potently increases the blood glucose level (4), whereas inactivation of this subset both impairs glucose counterregulation and blunts hyperglycemia induced by streptozotocin (5). Moreover, the activity of these neurons decreases rapidly following an increase in the blood glucose level, suggesting that, like pancreatic β cells, these neurons are capable of detecting and responding to changes in glycemia (6).

Another hypothalamic area implicated in glucose homeostasis is the paraventricular nucleus (PVN). Like the VMN, this brain area plays a key role in autonomic regulation, and contained within it are several distinct neuronal subpopulations that have been shown to influence circulating levels of insulin, glucose, or both (7–9). Among these is a subset of PVN neurons marked by expression of tyrosine hydroxylase (TH) (PVN^TH^ neurons) (10) that have been implicated in fuel homeostasis (11). In the current work, we hypothesized that these neurons, like neurons in the VMN, participate in autonomic control of glucose homeostasis. The current studies were undertaken to investigate this hypothesis.

Best known for its role in controlling blood pressure and cardiovascular function, the renin-angiogenin system (RAS) is also implicated in the control of glucose homeostasis. For example, excessive RAS activity is associated with obesity and insulin resistance in humans (12, 13), and it has been suggested to play a causal role in obesity-associated metabolic impairment (14, 15). One potential mechanism that might contribute to these effects is excessive RAS activity in the brain, which can mediate deleterious effects by activating neuronal (pro)renin receptors (PRRs) (16, 17). Upon binding to either of its ligands (prorenin or renin) (18–20), the PRR generates angiotensin II (Ang II) while also functioning as a tyrosine kinase that can engage multiple intracellular signal transduction cascades. Interestingly, whereas renin circulates at normal or low levels in patients with obesity and diabetes (21–23), plasma levels of prorenin are elevated in this population (23–28); in a mouse model of diet-induced obesity (DIO), prorenin levels are elevated in the hypothalamus as well as in plasma (29). Moreover, targeted deletion of neuronal PRR was recently shown to ameliorate glucose metabolic impairment in these animals (29).

The goal of the current work is to identify neuronal subsets upon which PRR signaling acts to impair glucose homeostasis. Based on our finding that the vast majority of PVN^TH^ neurons express the PRR, we focused on the specific question of whether PRR activity in these neurons is linked to obesity-associated metabolic impairment. We report that (a) the PRR is expressed by a large percentage of PVN^TH^ neurons, (b) PRR activation potently inhibits these neurons, (c) chemogenetic inhibition of PVN^TH^ neurons mimics the effect of a high-fat diet (HFD) to impair glucose homeostasis, and (d) selective deletion of the PRR from these neurons ameliorates the effect of DIO on both the circulating glucose level and glucose tolerance. Since these effects occurred in the absence of significant differences in body weight, our data suggest that, in normal animals, PVN^TH^ neurons play a physiological role in promoting normal glucose homeostasis — independently of the control of body weight — and that inhibition of these neurons by increased PRR signaling contributes to the effect of obesity to impair glucose homeostasis.

## Results

### Expression of the PRR and vasopressin by PVN^TH^ neurons in mice.

**Figure 1:**
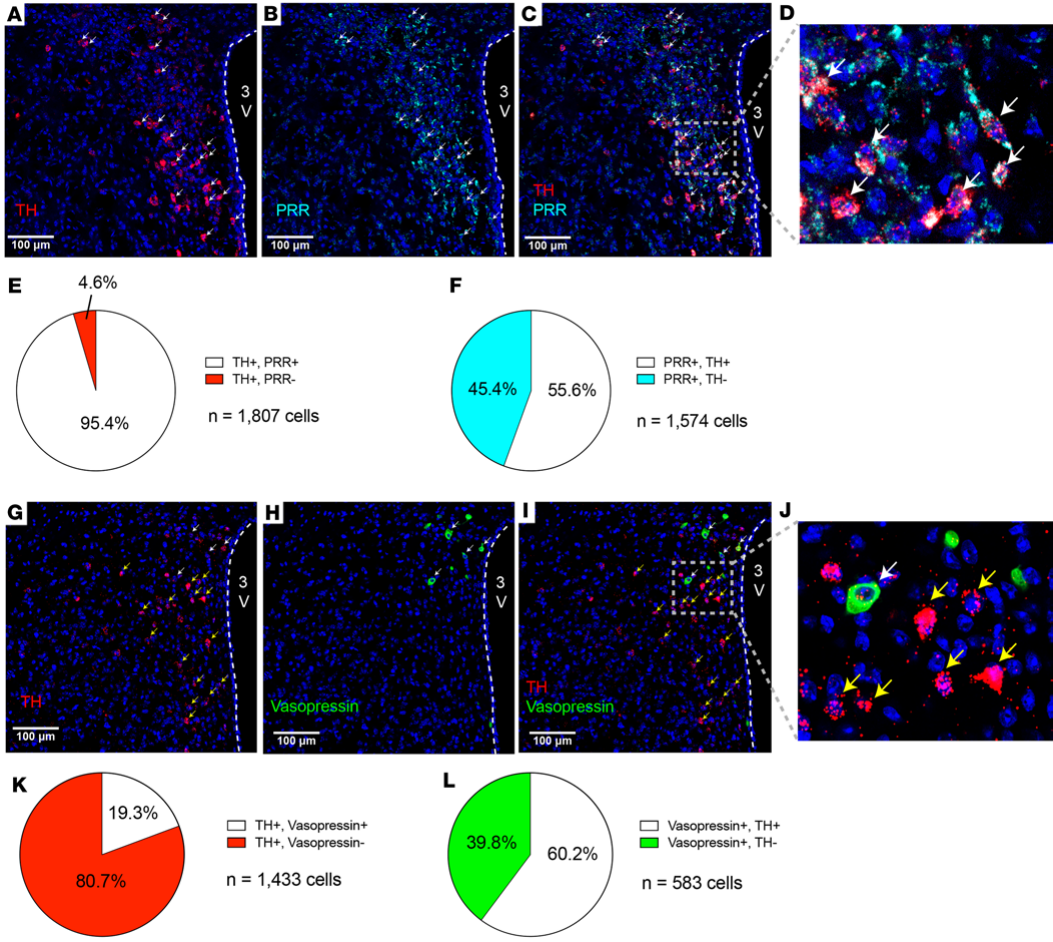
Expression of (pro)renin receptor and vasopressin in the PVN^TH^ neurons. (**A** and **B**) Representative images of RNAScope in situ hybridization of Th (red), Prr (cyan), and DAPI (blue) in the mouse PVN (bregma, –0.82 mm). (**C**) Merged image showing colocalization of Th and Prr mRNA in the PVN. (**D**) Digital enlarged views of the gray boxed areas in **C**. White arrows indicate cells expressed both Th and Prr mRNA. (**E** and **F**) Quantification of Th and Prr colocalization in the PVN (*n* = 6 mice, **E**; *n* = 3 mice, **F**). (**G** and **H**) Representative images of RNAScope in situ hybridization of Th (red), vasopressin (green), and DAPI (blue) in the mouse PVN (bregma, –0.88 mm). (**I**) Merged image showing colocalization of Th and vasopressin mRNA in the PVN. (**J**) Digital enlarged views of the gray boxed areas in **I**. White arrows indicate cells expressed both Th and vasopressin mRNA. Yellow arrows indicate cells expressed Th mRNA without vasopressin mRNA. (**K** and **L**) Quantification of TH and vasopressin colocalization in the PVN (*n* = 3 mice). PVN slices from bregma –0.58 mm to –1.06 mm were used. Scale bars: 100 μm.

*Prr* mRNA and PRR protein are expressed by neurons in both the rodent and human brain (30, 31), including presympathetic and vasopressin-containing neurons in the PVN (32, 33). To determine if either PRR or vasopressin are expressed in PVN^TH^ neurons, we used RNAScope in situ hybridization. Negative controls detecting the bacterial gene DapB were included to exclude nonspecific/background staining (Supplemental Figure 1; supplemental material available online with this article; https://doi.org/10.1172/jci.insight.174294DS1). We report that, in the PVN, the vast majority of *Th* mRNA expression is colocalized with Prr mRNA (Figure 1, A–D). Thus, quantitative analysis of 1,807 TH neurons throughout the PVN (bregma, –0.58 mm to –1.06 mm) revealed that approximately 95% of the PVN^TH^ neurons express the Prr mRNA (Figure 1E). By comparison, only 56% of Prr-expressing neurons coexpress *Th* mRNA (Figure 1F). We also report that only 19% of 1,433 TH^+^ PVN neurons coexpress vasopressin (bregma, –0.58 mm to –1.06 mm) (Figure 1, G–K), although 60% of vasopressin^+^ neurons in this brain area coexpress *Th* mRNA (Figure 1L).

### Validation of PRR deletion from PVN^TH^ neurons.

**Figure 2:**
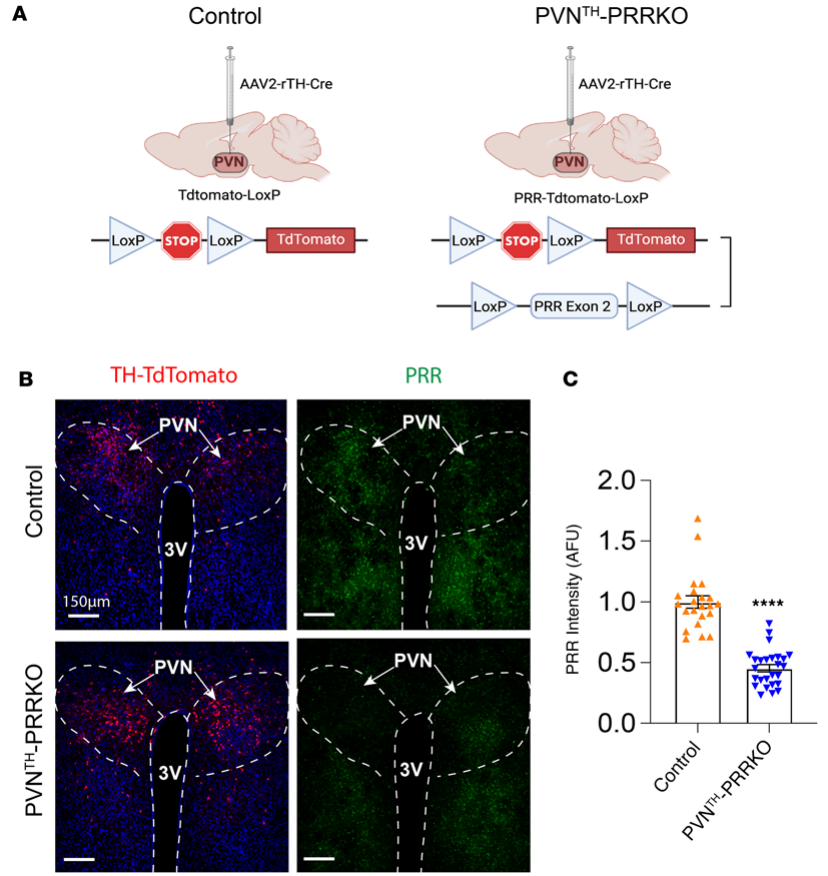
Validation of (pro)renin receptor (PRR) knockdown efficiency in PVN^TH^ neurons. (**A**) Schematic representation of Cre-LoxP–mediated deletion of PRR in PVN^TH^ neurons in tdTomato-LoxP (tdTomato^fl/fl^) or PRR-tdTomato-LoxP mice. AAV-expressing Cre recombinase driven by a rat tyrosine hydroxylase promoter (AAV2-rTH-Cre) was used to delete PRR and induce tdTomato reporter expression in mice bearing both PRR-LoxP and tdTomato-LoxP alleles, shown as PVN^TH^-PRRKO. The tdTomato-loxP mice received the AAV2-rTH-Cre to the PVN and were used as a control. (**B**) Representative images showing TH-tdTomato (red), DAPI (blue), and PRR immunofluorescence (green) in the PVN (bregma, –1.06 mm). Scale bar: 150 μm. (**C**) Quantification data of PRR immunoreactive intensity. Data are expressed as mean ± SEM. ****P < 0.0001, unpaired Student’s *t* test. (*n* = 3 animals/group, 22–26 PVNs/group).

Having established that most PVN^TH^ neurons express PRR, we next sought to investigate the role played by PRR signaling by deleting the PRR specifically from PVN^TH^ neurons. This goal was achieved by bilateral microinjection of a viral vector that expresses cre recombinase under the control of the rat TH promoter (AAV2-rTH-Cre) into the PVN. To enable visualization of virally mediated recombination in TH-expressing neurons, double-floxed mice in which both the PRR allele and a STOP cassette for tdTomato are flanked by loxP sites (PRR-tdTomato-LoxP) and their controls (loxP-flanked-STOP tdTomato allele only; tdTomato-LoxP; Control) were studied (Figure 2A). Following the microinjection of AAV2-rTH-Cre into the PVN of these mice, tdTomato expression was induced specifically in TH-expressing neurons (Figure 2B, left panel), and PRR deletion from these neurons (PVN^TH^-PRRKO) was confirmed 1 month after microinjection by immunofluorescence labeling (Figure 2, B and C). These data indicate that PRR deletion was successful in just half of PVN^TH^ neurons. We anticipate that, in future studies, the phenotype induced by PVN^TH^ neuron–specific KO will be more robust if achieved in a larger fraction of these neurons.

### Effect of selective deletion of PRR from PVN^TH^ neurons on glucose homeostasis.

**Figure 3:**
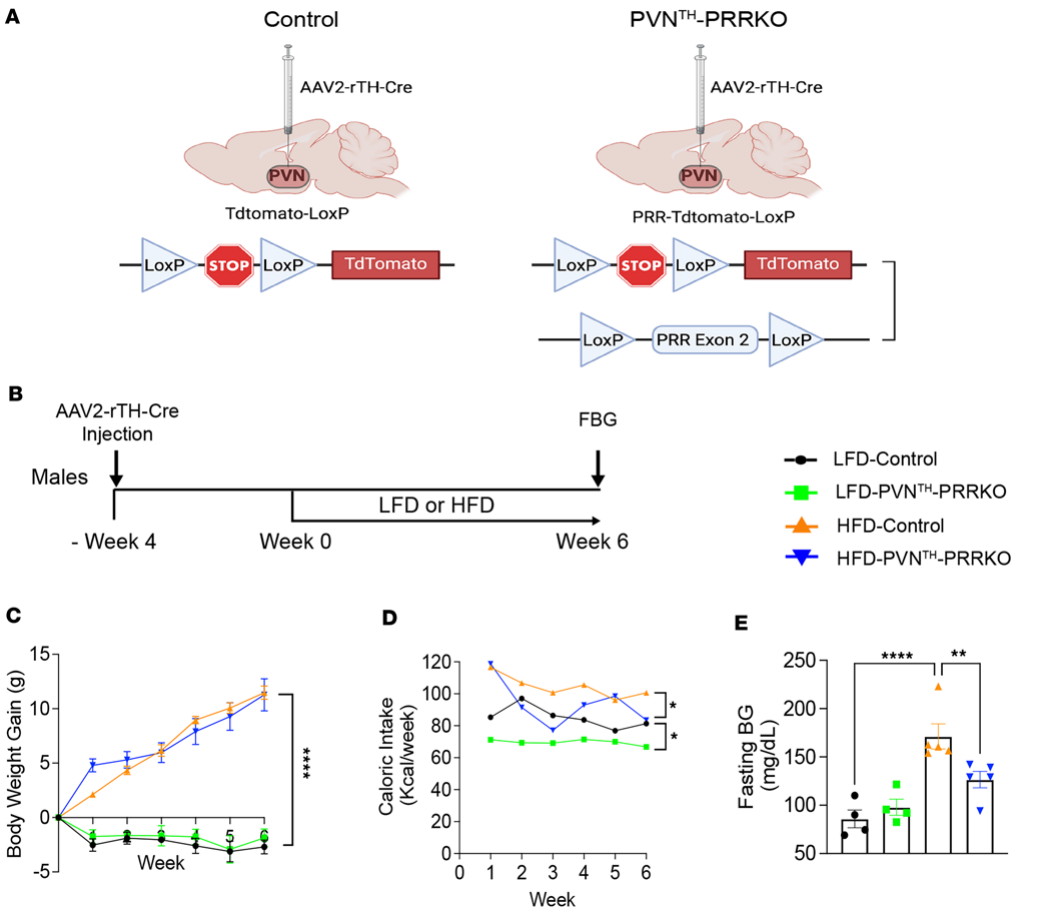
Deletion of (pro)renin receptor (PRR) in PVN^TH^ neurons lowers fasting blood glucose (FBG) in HFD-induced obese male mice. (**A**) Schematic representation of Cre-LoxP–mediated deletion of PRR in PVN^TH^ neurons in PRR-tdTomato-LoxP mice. Mice bearing both PRR-LoxP and tdTomato-LoxP alleles (PRR-tdTomato-LoxP) received bilateral microinjection directed to the PVN of an AAV-expressing Cre recombinase driven by a rat tyrosine hydroxylase promoter (AAV2-rTH-Cre), while tdTomato-LoxP mice received the AAV2-rTH-Cre virus to the PVN and were used as controls. (**B**) Schematic diagram of the experimental protocol. Four weeks after viral injection, mice were placed on either a low-fat diet (LFD, 10% calories from fat) or a high-fat diet (HFD, 60% calories from fat). (**C**–**E**) Weekly body weight gain, calorie intake, and fasting blood glucose at 6 weeks following exposure to either an HFD or an LFD. *n* = 4–5 animals/group. Data are expressed as mean ± SEM. *P < 0.05, **P < 0.01, ****P < 0.0001. Two-way ANOVA with Fisher’s LSD multiple comparisons tests were used.

Using this approach (also illustrated in Figure 3A), PVN^TH^-PRRKO and their controls were allowed 4 weeks to recover from and to allow for viral expression after adeno-associated virus (AAV) microinjection. Mice were then placed on either an HFD (60% Kcal from fat) or a matched, low-fat control diet (LFD) (10% Kcal from fat) for the next 6 weeks (Figure 3B). Male and female mice were studied separately.

In males, deletion of PRR in PVN^TH^ neurons in the LFD-fed group (LFD-PVN^TH^-PRRKO) had no significant effect on either body weight (Figure 3C) or fasting blood glucose (FBG) (Figure 3E), although calorie intake was slightly reduced (Figure 3D) relative to LFD-Control. As expected, the 6-week HFD regimen increased body weight (Figure 3C), calorie intake (Figure 3D), and FBG levels (Figure 3E) in controls (HFD-Control) compared with their LFD-fed counterparts (LFD-Control). Despite similar increases in body weight (Figure 3C), FBG levels were significantly lower in HFD-PVN^TH^-PRRKO mice than in HFD-control mice (Figure 3E).

To confirm these findings and to detect any background- or strain-dependent effects of PVN^TH^-PRRKO, we performed a complementary study in a strain of male mice bearing the floxed PRR allele but without the floxed tdTomato reporter (PRR^fl/y^ mice). Following bilateral microinjection into the PVN of either a control virus (AAV2-TH-GFP) or AAV2-rTH-Cre to delete PRR in PVN^TH^ neurons into male PRR^fl/y^ mice (Supplemental Figure 2A), animals were given a 4-week recovery period and to allow for AAV2 expression. These 2 groups of mice were then placed on either the HFD or LFD for 6 weeks, as above (Supplemental Figure 2B). In LFD-fed male mice, deletion of PRR from PVN^TH^ neurons once again had no significant effect on body weight (Supplemental Figure 2C), caloric intake (Supplemental Figure 2D), FBG (Supplemental Figure 2E), or glucose tolerance (Supplemental Figure 2, F–H). Compared with their lean counterparts, the 6-week HFD regimen in male mice resulted in the expected increase of body weight (Supplemental Figure 2C), calorie intake (Supplemental Figure 2D), and FBG levels (Supplemental Figure 2E), along with impaired glucose tolerance (Supplemental Figure 2, F–H). Despite exhibiting similar increases in body weight and calorie intake as HFD-Control mice (Supplemental Figure 2, C and D), HFD-PVN^TH^-PRRKO mice once again had lower FBG levels (Supplemental Figure 2E) and improved glucose tolerance (Supplemental Figure 2H) compared with HFD-control mice. These data confirm that, in male DIO mice, the beneficial effect of PRR deletion in the PVN^TH^ neurons is evident irrespective of the presence of the tdTomato reporter.

To evaluate the possibility of a sex effect on this phenotype, we repeated the above studies in female mice (either with or without a tdTomato reporter). As a first step, PRR^fl/fl^ mice received a bilateral microinjection into the PVN of either a control virus (AAV2-TH-GFP) or AAV2-rTH-Cre to delete PRR in PVN^TH^ neurons (Supplemental Figure 3A) and were placed on LFD or HFD after 4 weeks of recovery (Supplemental Figure 3B). In LFD-fed female mice, PRR deletion from PVN^TH^ neurons had no significant effect on FBG (Supplemental Figure 3C) or glucose tolerance (Supplemental Figure 3, D–F), as expected. Exposure to the HFD for 6 weeks significantly elevated FBG (Supplemental Figure 3C) but did not impair glucose tolerance in these female mice (Supplemental Figure 3, D–F) relative to LFD-Control; unlike in males, PRR deletion from PVN^TH^ neurons had no effect on either FBG (Supplemental Figure 3C) or glucose tolerance (Supplemental Figure 3, D–F) relative to controls. We also observed no effect of selective deletion of PRR from PVN^TH^ neurons on either body weight or food intake in either LFD or HFD-fed mice.

To confirm these findings in females, and to examine whether the effect of PVN^TH^-PRRKO is background or strain dependent, the studies were repeated in female PRR-tdTomato-LoxP mice and female tdTomato-LoxP mice controls (Supplemental Figure 4, A and B). As was observed in female PRR^fl/fl^ mice, deletion of PRR from PVN^TH^ neurons affected neither FBG (Supplemental Figure 4C) nor glucose tolerance in female mice fed the LFD (Supplemental Figure 4, D–F). Similarly, consuming the HFD for 6 weeks raised the FBG (Supplemental Figure 4C) but did not significantly impair glucose tolerance (Supplemental Figure 4, D–F) relative to female LFD-Control, and once again, deletion of PRR in PVN^TH^ neurons in female mice had no effect on FBG (Supplemental Figure 4C) or glucose tolerance (Supplemental Figure 4, D–F) fed a HFD, unlike what was observed in males. Together, these findings suggest PPR signaling in PVN^TH^ neurons exerts a sexually dimorphic effect, with glucose homeostasis being improved in male but not female HFD-fed mice.

### Effect of selective deletion of PRR from PVN^TH^ neurons on cardiovascular function and locomotor behavior.

To determine the effect of PRR deletion in PVN^TH^ neurons on cardiovascular parameters, telemetry transmitters were implanted into male mice to continuously monitor blood pressure, heart rate, and locomotor activity in freely moving conditions described previously (31, 34). As described earlier, both tdTomato-LoxP and PRR-tdTomato-LoxP mice underwent bilateral injection of AAV2-rTH-Cre into the PVN (Supplemental Figure 5A). Following telemetry transmitter implantation and recovery, cardiovascular parameters were recorded continuously at baseline and during 6 weeks of HFD treatment, as illustrated in Supplemental Figure 5B. At baseline (week 0), when mice were fed a regular chow diet, neither mean arterial blood pressure (Supplemental Figure 5, C and D) nor heart rate (Supplemental Figure 5, E and F) or locomotor activity (Supplemental Figure 5, G and H) differed between Control and the PVN^TH^-PRRKO mice. Within 1 week after switching to the HFD, mean arterial blood pressure (Supplemental Figure 5, C and D) and heart rate (Supplemental Figure 5, E and F) were increased during both the light cycle and dark cycle in all mice, and this effect persisted throughout the dietary regimen. However, these cardiovascular responses to the HFD were not significantly affected by PRR deletion from PVN^TH^ neurons (Supplemental Figure 5, B–G). Similarly, no differences in locomotor activity were observed in response to the HFD in either group (Supplemental Figure 5, G and H). Collectively, these findings show that, in male mice, the deleterious effects of HFD on glucose homeostasis, but not on body weight, blood pressure, or heart rate, were ameliorated by PRR deletion, specifically from PVN^TH^ neurons.

### Effects of PRR signaling on insulin secretion, insulin sensitivity, and insulin-independent glucose disposal.

To explore the peripheral mechanisms underlying the beneficial effects on glucose homeostasis, we performed a frequently sampled i.v. glucose tolerance test (FSIGT) on male DIO mice with or without PRR deletion from PVN^TH^ neurons. This method was chosen because, unlike other phenotyping methods, the FSIGT quantifies both glucose tolerance and glucose-induced insulin secretion (acute insulin response to glucose AIR_G_]) while also deriving validated estimates (35) of both insulin sensitivity index (S_I_) and insulin-independent glucose uptake (S_G_) from minimal model analysis of plasma insulin and glucose levels.

**Figure 4:**
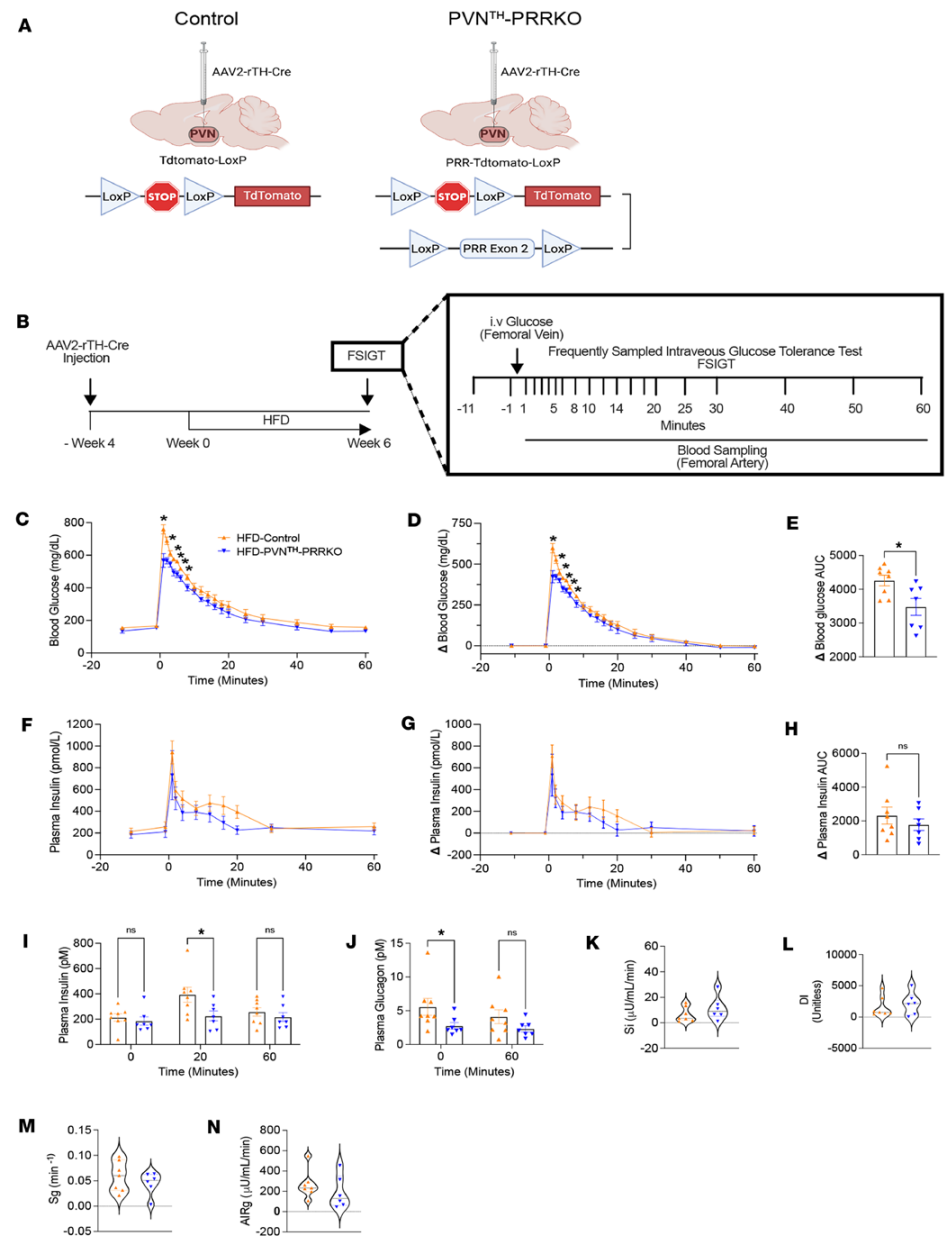
Effects of (pro)renin receptor (PRR) deletion in PVN^TH^ neurons on determinants of glucose tolerance. (**A**) Schematic representation of Cre-LoxP–mediated deletion of PRR in PVN^TH^ neurons in PRR-tdTomato-LoxP mice. AAV-expressing Cre recombinase driven by a rat tyrosine hydroxylase promoter (AAV2-rTH-Cre) was used to delete PRR and induce tdTomato reporter expression in mice bearing both PRR-LoxP and tdTomato-LoxP alleles, shown as PVN^TH^-PRRKO. The tdTomato-loxP mice that received the AAV2-rTH-Cre were used as controls. (**B**) Schematic diagram of the experimental protocol. Mice received a high-fat diet (HFD, 60% calories from fat) for 6 weeks. (**C**–**E**)Blood glucose levels, changes in blood glucose levels, and glucose AUC from baseline during the FSIGT. (**F**–**H**) Plasma insulin levels, changes in plasma insulin levels from baseline, and respective insulin AUC during FSIGT. (**I** and **J**) Plasma insulin levels (t = 0, 20, 60 min) and glucagon levels (t = 0, 60 min) during the FSIGT. (**K**–**N**) Insulin sensitivity (S_I_), deposition index (D_I_), glucose effectiveness (S_G_), and acute insulin response to glucose (AIR_G_) were analyzed from plasma insulin and glucose profiles during FSIGT using MINMOD Millennium software. *n* = 6-7 mice/group. Data are expressed as mean ± SEM. *P < 0.05, 2-way ANOVA followed by Fisher’s LSD tests for **D**, **F**, **G**, **I**, and **J**; unpaired *t* test for **E** and **H**; unpaired Mann-Whitney test for **K**–**N**.

Studies were performed following bilateral injection to the PVN of an AAV2-rTH-Cre virus in male mice bearing either the PRR-floxed allele and the loxP-flanked STOP cassette for tdTomato or only the loxP-flanked STOP cassette for tdTomato (Figure 4A), as described above. Mice were then subjected to an FSIGT after consuming the HFD for 6 weeks and undergoing arterial and venous catheterization (Figure 4B). As predicted, glucose tolerance was improved in male HFD-PVN^TH^-PRRKO mice compared with DIO-Controls (Figure 4, C–E). Surprisingly, plasma insulin levels tended to be lower in the HFD-PVN^TH^-PRRKO mice than in HFD-Controls, although this effect did not achieve statistical significance (except at the 20-minute mark after glucose injection; Figure 4, F–I). Nevertheless, combined with the finding of no difference in the acute insulin response to glucose (AIR_G_) between the 2 groups, we infer that improved glucose tolerance in PRR-deleted mice is not due to increased insulin secretion. By comparison, fasting plasma glucagon levels were significantly reduced by PRR deletion PVN^TH^ neurons from (Figure 4J), which could have played a role by reducing hepatic glucose production (36, 37).

Minimal model analysis of plasma glucose and insulin levels obtained during the FSIGT (35, 38, 39) did not identify statistically significant differences between the 2 groups in any parameter. Nevertheless, the tendency for the S_I_ to be higher in HFD-fed mice in which PRR was deleted from PVN^TH^ neurons than in controls (Figure 4K) suggests that increased insulin sensitivity could have contributed to the observed improvement of glucose tolerance, and additional studies are warranted to test this hypothesis. In addition, neither the disposition index (D_I_) nor glucose effectiveness (S_G_; a measure of insulin-independent glucose disposal) (Figure 4, L–N) differed significantly between groups. These findings suggest that, in DIO mice, PRR signaling in PVN^TH^ neurons impairs glucose tolerance via a mechanism that, while not involving decreased insulin secretion, may involve reduced insulin sensitivity. Additional studies are warranted to test this hypothesis.

### PVN^TH^ neurons are inhibited by PRR activation.

**Figure 5:**
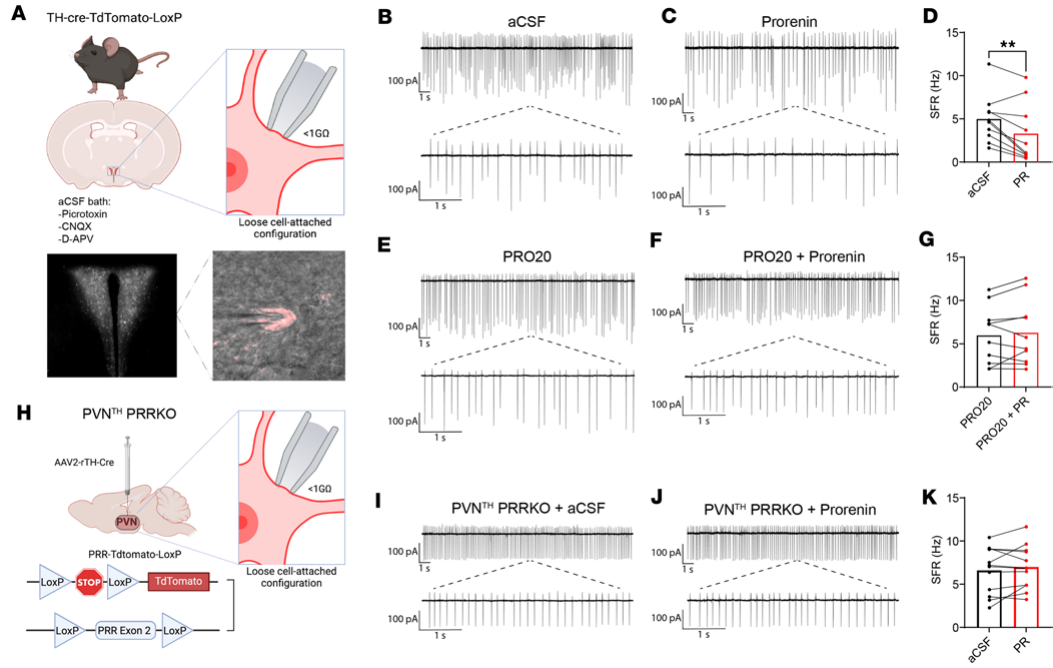
Prorenin decreases the spontaneous firing rate of PVN^TH^ neurons. (**A**) Experimental illustration of a recorded PVN^TH^ neuron under loose cell-attached configuration in the presence of blockers of synaptic transmission. Picrotoxin, 50 μM; antagonist of AMPA/kainate receptors, CNQX, 5 μM; NMDA receptor antagonist, D-APV, 50 μM. (**B** and **C**) Representative raw traces of loose cell-attached recordings of spontaneous firing activity in a PVN^TH^ neuron in aCSF or 2.5 nM prorenin (PR). (**D**) Summary data of spontaneous firing rate (SFR) showing that application of PR decreased SFR of PVN^TH^ neurons (*n* = 7 mice/10 neurons). (**E** and **F**) Representative raw traces of loose cell-attached recordings of spontaneous firing activity in a PVN^TH^ neuron in the presence of 250 nM PRO20, a specific (pro)renin receptor (PRR) antagonist, and PRO20 + PR. (**G**) Summary data of SFR of PVN^TH^ neurons in the presence of PRO20 (*n* = 7 mice/10 neurons). (**H**) Schematic representation of Cre-LoxP–mediated deletion of PRR in PVN^TH^ neurons in PRR-tdTomato-LoxP mice. (**I** and **J**) Representative raw traces of loose cell-attached recordings of spontaneous firing activity in a PVN^TH^ neuron from a PVN^TH^ PRRKO mouse. (**K**) Summary data of SFR showing that PR did not affect the SFR of PVN^TH^ neurons in PVN^TH^ PRRKO mice (*n* = 6 mice/12 neurons). Data are obtained from both male and female mice and are expressed as mean ± SEM. **P < 0.01, 2-tailed paired Student’s *t* test was used.

To better understand cellular mechanisms through which PRR signaling influences PVN^TH^ neurons, we performed slice electrophysiology on TH^+^ neurons identified in the PVN of both male and female TH-Cre–driven tdTomato reporter mice. Specifically, cell-attached patch-clamp electrophysiology was used to measure the effect of the application of the PRR agonist prorenin on PVN^TH^ neuron activity. To exclude any potential effect of prorenin on neurons lying upstream, these studies were performed in the presence of synaptic transmission blockers (Figure 5A). We found that, following bath application of prorenin, the spontaneous firing rate (SFR) of PVN^TH^ neurons was reduced by ~30% compared with artificial cerebrospinal fluid (aCSF) control (Figure 5, B–D). To validate the specificity of this effect, we repeated this study in both the presence and absence of PRO20, a specific PRR antagonist (19). Our finding that PRO20 fully blocked the effect of prorenin on PVN^TH^ neuron firing rate (Figure 5, E–G) indicates that PRR activation is required for the inhibitory effect of prorenin on PVN^TH^ neurons. Experiments conducted on brain slices from mice lacking PRR, specifically in the PVN^TH^ neurons, yielded similarly negative findings (Figure 5, H–K), confirming that the PRR must be expressed by PVN^TH^ neurons for these neurons to be inhibited by prorenin. Future studies are needed to identify the signal transduction mechanisms underlying this inhibitory effect.

### Chemogenetic inhibition of PVN^TH^ neurons elevates blood glucose in mice.

**Figure 6:**
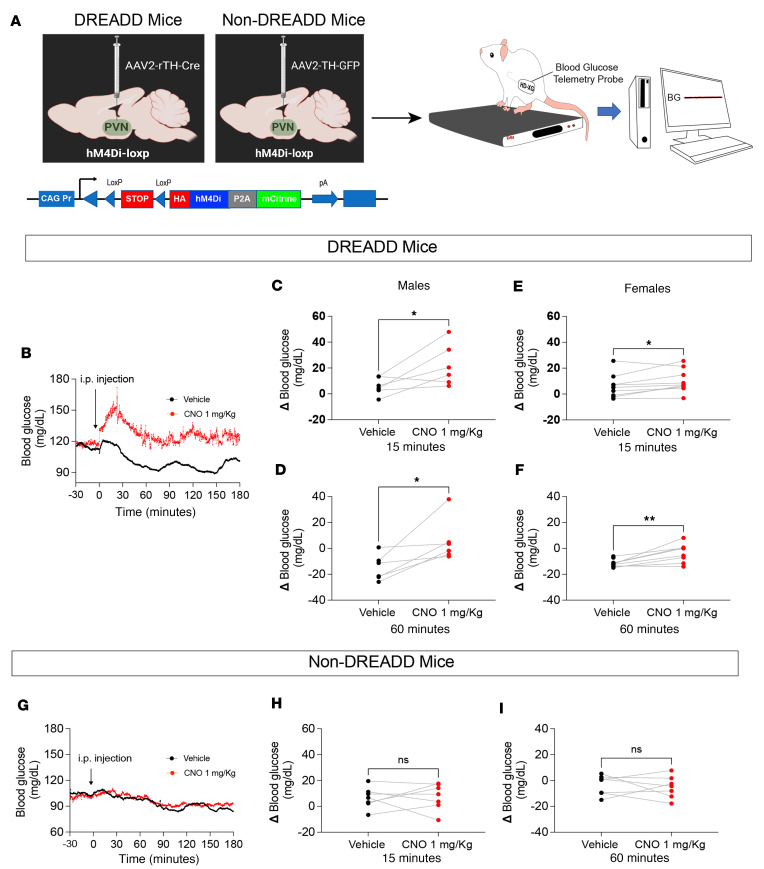
Chemogenetic inhibition of PVN^TH^ neurons increases blood glucose in both male and female mice. (**A**) Schematic diagram of 1-time injections of either AAV2-TH-GFP (control virus, non-DREADD) or AAV2-rTH-Cre (DREADD) into the PVN of hM4Di-LoxP mice. One month after the AAV2 injection, mice were implanted with HD-XG telemetric glucose transmitters followed by continuous glucose monitoring (CGM). (**B**) Representative traces of CGM following injection of either vehicle or clozapine N-oxide (CNO, 1 mg/Kg) for 3 hours in DREADD mice. (**C** and **D**) Summary data of blood glucose levels following injection of either vehicle or CNO at 15 and 60 minutes in male mice (*n* = 6). (**E** and **F**) Summary data of blood glucose levels following injection of either vehicle or CNO at 15 and 60 minutes in female mice (*n* = 9). (**G**) Representative traces of CGM following injection of either vehicle or clozapine N-oxide (CNO, 1 mg/Kg) for 3 hours in non-DREADD mice. (**H** and **I**) Summary data of blood glucose levels following injection of either vehicle or CNO at 15 and 60 minutes in both male (*n* = 3) and female (*n* = 4) non-DREADD mice. Data are expressed as mean ± SEM. *P < 0.05, **P < 0.01, 2-tailed paired Student’s *t* test was used.

Since selective deletion of the PRR from PVN^TH^ neurons blocks the deleterious effect of DIO on glucose homeostasis, and since PVN^TH^ neurons are inhibited by PRR activation, we next tested the hypothesis that, in mice fed standard chow, chemogenetic inhibition of PVN^TH^ neurons would mimic the deleterious effect of DIO to impair glucose homeostasis. To this end, we combined designer receptors exclusively activated by designer drugs (DREADD) technology with continuous telemetric glucose monitoring in both male and female mice engineered to express inhibitory human M4 muscarinic receptors (hM4Di) on TH-expressing neurons. For the non-DREADD control group, non-Cre (GFP) virus–infected mice were used (Figure 6A).

Upon i.p. administration of saline vehicle, a rapid but transient elevation of blood glucose levels was observed, presumably the result of a nonspecific stress response. In contrast, i.p. administration of the DREADD receptor agonist CNO (1 mg/kg) elicited a notable elevation of blood glucose levels that persisted throughout the 3-hour monitoring period (compared with controls) in both male and female mice (Figure 6, B–F). Consistent with our earlier findings, we observed that, when segregating data by sex, male mice (Figure 6, C and D) exhibited a much greater glycemic elevation than females in response to PVN^TH^ neuron inhibition (Figure 6, E and F). A similar set of responses was observed in the DIO model. To exclude the possibility that this increase in glycemia was due to nonspecific CNO effects (unrelated to PVN^TH^ neuron inhibition), we replicated the experiment in non-DREADD control mice. While the initial, transient rise of blood glucose was once again observed following the i.p. injection of either vehicle or CNO (Figure 6G), no sustained elevation of glycemia was observed in response to CNO administration (Figure 6, H and I). Collectively, these data establish that chemogenetic inhibition of PVN^TH^ neurons raises the blood glucose level.

### PVN^TH^ neurons project to autonomic and neuroendocrine regulatory brain regions.

**Figure 7:**
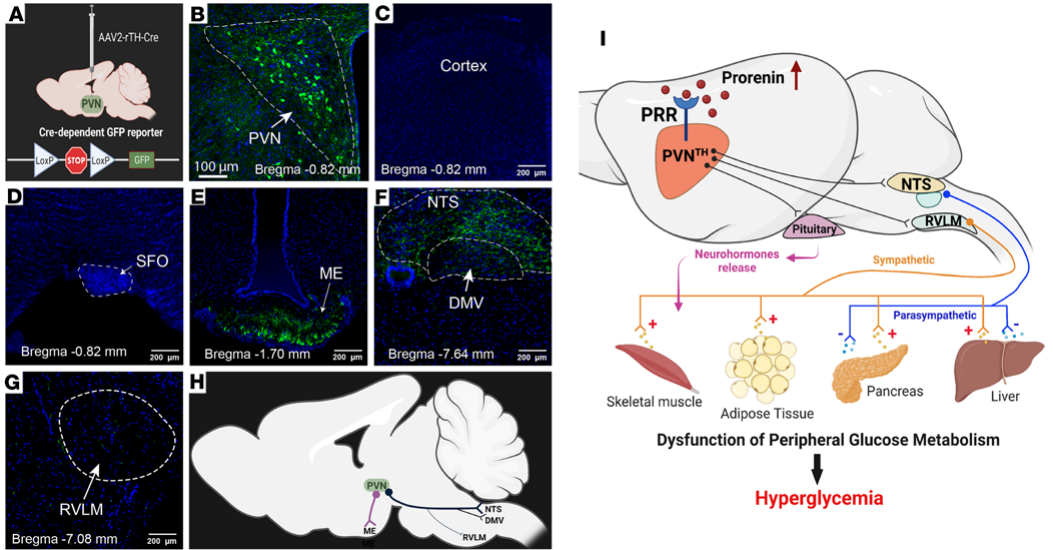
Anatomical mapping of tyrosine hydroxylase neurons (PVN^TH^) in the hypothalamic paraventricular nucleus and the working hypothesis. (**A**) Schematic representation of Cre-mediated deletion Flox-STOP in Cre-dependent GFP^fl/fl^ mice. AAV-expressing Cre recombinase driven by a rat tyrosine hydroxylase promoter (AAV2-rTH-Cre) was bilaterally injected into the PVNs of Cre-dependent GFP^fl/fl^ mice. (**B**–**G**) Representative images of PVN (**B**), brain cortex (**C**), subfornical organ (SFO; **D**), median eminence (ME; **E**), the nucleus of the solitary tract (NTS; **F**), the dorsal motor nucleus of the vagus (DMV; **F**), and the rostral ventrolateral medulla (RVLM; **G**). (**H**) A schematic summary of the PVN^TH^ neuronal projection map. The thicker lines represent denser projections, and the thinner lines represent lesser projections. *n* = 4 mice. (**I**) Working hypothesis. During diet-induced obesity, (pro)renin receptor (PRR) signaling activation in the tyrosine hydroxylase neurons in the hypothalamic paraventricular nucleus leads to inhibition of these PVN^TH^ neurons and, consequently, the dysregulation of peripheral glucose homeostasis by neural autonomic and/or neuroendocrine mechanisms.

To identify projections of PVN^TH^ neurons to brainstem autonomic regulatory nuclei, we microinjected AAV2-rTH-Cre into the PVN of Cre-dependent GFP reporter mice (Figure 7A), which labels both cell bodies and projecting fibers with GFP (Figure 7B). Whereas projections to the cerebral cortex (Figure 7C) or forebrain regions such as the subfornical organ (SFO) were not observed (Figure 7D), dense projections to the median eminence (ME; Figure 7E) and nucleus of the solitary tract (NTS; Figure 7F) were evident. To a lesser extent, projections to the dorsal motor nucleus of the vagus (DMV; Figure 7F) and rostral ventrolateral medulla (RVLM; Figure 7G) were also observed. Based on these findings, we generated a schematic that illustrates descending PVN^TH^ neuronal projections (Figure 7H), with thicker lines representing denser projections. Based on this anatomical evidence that TH^PVN^ neurons project to brain regions regulating endocrine and autonomic function (40–42), we hypothesize that, during DIO, impaired glucose tolerance and elevated FBG levels result at least in part from inhibition of PVN^TH^ neurons mediated by PRR activation (Figure 7I). As noted earlier, these effects occur independently of changes in body weight.

## Discussion

Our work demonstrates first that the PRR is expressed in the vast majority of PVN^TH^ neurons, and that PRR signaling exerts an inhibitory effect on these neurons. Using a chemogenetic approach, we further show that glucose homeostasis is impaired by inhibition of PVN^TH^ neurons in a manner resembling the effect of DIO, whereas deletion of the PRR, specifically from PVN^TH^ neurons, blunts the deleterious effect of DIO on glucose homeostasis. Furthermore, these effects are mediated via a mechanism that does not involve differences in body weight, is not associated with changes in heart rate or blood pressure, and appears to be specific for male mice. Together, these findings suggest that, in male mice, PVN^TH^ neuron activity is required for normal glucose homeostasis under physiological conditions and that inhibition of these neurons by increased PRR signaling contributes to obesity-associated impairment of glucose homeostasis.

The hypothalamic PVN comprises multiple neuronal subpopulations, including many neuroendocrine and preautonomic neurons that play critical roles in various physiological processes (43–45), including glucose homeostasis (46). Compared with PVN neurons involved in the control of the hypothalamic-pituitary-adrenal and hypothalamic-pituitary-thyroid axes (expressing CRH and TRH, respectively), as well as neurons expressing oxytocin or vasopressin, however, TH-containing neurons in this brain area have received limited attention. Most of these neurons appear to be both GABAergic and dopaminergic (10), and they have been suggested to participate in the control of sympathetic activity to brown adipose tissue (BAT) (11). Their role in glucose homeostasis, however, remains unexplored.

Based on our finding that the blood glucose level is rapidly elevated following their chemogenetic inhibition, PVN^TH^ neurons join the growing list of hypothalamic neurons implicated as contributors to the biologically defended level of glycemia (3, 46). We also present electrophysiological evidence that PRR activation exerts a direct, inhibitory effect on PVN^TH^ neurons that does not require synaptic transmission. Combined with our finding that the deleterious effects of DIO on both the circulating glucose level and glucose tolerance are ameliorated by PRR deletion from PVN^TH^ neurons, we infer a role for PRR signaling in these neurons in the pathogenesis of obesity-associated glucose metabolic impairment. Interestingly, this effect occurs independently of changes in food intake, body weight, blood pressure, or heart rate, suggesting a high degree of functional specificity. Questions that remain to be addressed include whether experimental activation of PVN^TH^ neurons can blunt the effect of obesity to impair glucose tolerance and the ways in which PRR signaling and the activity of these neurons are affected by DIO.

The PRR (18) is an essential component of the brain RAS that regulates blood pressure, heart rate, and other cardiovascular functions (16, 17). In addition to the local generation of Ang II, which binds to and activates neuronal angiotensin receptors, binding of the PRR by either of its 2 endogenous ligands (prorenin or renin) activates an intracellular tyrosine kinase signal transduction cascade (16, 47). Interestingly, while activation of neuronal PRR signaling has been shown to promote hypertension development by increasing vasopressin release (30, 32, 48, 49), we report that only a small percentage of PVN^TH^ neurons express vasopressin. Indeed, our work suggests little, if any, role of PRR signaling in PVN^TH^ neurons in cardiovascular regulation.

Recently, a role for brain RAS activity in the control of energy balance has been suggested (14, 15). Specifically, intracerebroventricular (ICV) administration of Ang II reduces food consumption, body weight, and adipose tissue mass while simultaneously increasing thermogenesis in BAT, lipolysis in white adipose tissue (WAT), and overall energy expenditure in rats (50, 51). Deletion of Ang II type 1a receptor (AT_1a_R), specifically in leptin receptor–expressing neurons, also inhibits the increase of resting metabolic rate that occurs as a response to an HFD (52). Interestingly, we showed that PRR deletion in the PVN^TH^ neurons does not affect food intake or body weight; instead, it attenuates the glucose metabolic impairment induced by consuming an HFD. Whether this effect involves the local generation of Ang II awaits further study; the data together are suggestive of brain region- or cell type–specific effects of brain RAS across various aspects of fuel homeostasis.

Interestingly, we observed that PRR deletion in the PVN^TH^ neurons does not improve the glucose metabolic phenotype in female mice. Moreover, although chemogenetic inhibition of PVN^TH^ neurons raised the blood glucose level in both sexes, the effect was much smaller in females (~4 mg/dL) than in their male counterparts (~17 mg/dL). Together, these findings suggest a more prominent role of PVN^TH^ neurons in the control of glucose homeostasis in males. Interestingly, however, ex vivo electrophysiological analysis did not reveal a sex difference in the inhibitory effect of PRR activation on PVN^TH^ neuron activity. Thus, we infer that the sex difference is not localized to a failure of PVN^TH^ neurons to respond to PRR activation in females; more likely, the sex difference in phenotype involves circuits lying downstream of these neurons. Given that CNS control of glucose homeostasis involves a complex network of glucoregulatory circuits, it is perhaps unsurprising that this system can be modulated by variables including sex. In this context, we note that male mice tend to be more prone to obesity-associated metabolic impairment than females; future studies that focus on the identification of these downstream neurocircuits will eventually permit investigation into whether they are regulated by PRR signaling in PVN^TH^ neurons in a sex-dependent manner.

While the peripheral mechanism whereby PRR deletion from PVN^TH^ neurons lowers blood glucose and improves glucose tolerance in male mice remains uncertain, our work excludes increased insulin secretion as a key factor. More likely is that the effect involves increased insulin sensitivity, possibly via changes in autonomic outflow and HPA axis activity secretion, in addition to our finding of reduced glucagon levels, expected to lower hepatic glucose production (36, 37). Future studies to investigate whether increased PVN^TH^ neuron activity inhibits glucagon secretion from pancreatic α cells (4, 38, 53) will be of interest.

One limitation of the current studies is that, while all animals received bilateral PVN injections, a small subset of mice was “hit” unilaterally, there were differences in the spread of viral injection throughout the rostral-caudal extent of the PVN, and there was a small amount of spillover, targeting TH neurons near the PVN. Thus, we cannot exclude a potential contribution of our observations made by these TH neurons. However, our findings were consistent across multiple studies, supporting the conclusions drawn on the effect of PRR, although future studies are warranted to identify the distinct subset of PVN^TH^-PRR neurons that mediate the observed phenotype. In summary, our data identify PVN^TH^ neurons as participants in CNS regulation of glucose homeostasis and implicates PRR-mediated inhibition of these neurons in the pathogenesis of obesity-associated glucose metabolic impairment.

## Methods

### Sex as a biological variable.

Both male and female mice were used in the study.

### Animals.

Mice in a C57BL/6J genetic background were used in this study, except for TH^Cre+^ and hM4Di^fl/fl^ mice, which are in a C57BL/6NJ background. The C57BL/6J mice (stock no. 000664), GFP^fl/fl^ mice (stock no. 030220) (54), TH^Cre/+^ mice (stock no. 008601) (55), tdTomato^fl/fl^ (stock no. 007914) (56), and human muscarinic receptor 4 coupled to Gi protein hM4Di^fl/fl^ (stock no. 026219) (57) were purchased from The Jackson Laboratory. The PRR-LoxP (male, PRR^fl/y^; female, PRR^fl/fl^) mice were maintained in the Feng Earley laboratory (University of Nevada, Reno) (20). The PRR-LoxP mice were crossed with the tdTomato^fl/fl^ for a few generations to generate double-LoxP mice for PRR and tdTomato (PRR-tdTomato-LoxP). Hemizygous TH^Cre+^ mice were crossed with tdTomato^fl/fl^ mice to generate reporter mice that express tdTomato in TH^+^ cells (TH^Cre+^tdTomato^fl/+^).

All mice were maintained in the Animal Care Facility at the University of Nevada, Reno, under a 12-hour light-dark cycle and room temperature of 21°C–23°C with ad libitum access to a standard chow diet (Envigo, catalog 2019) before other diet regiments.

### RNAScope in situ hybridization.

In situ hybridization of TH and PRR mRNAs was performed using an RNAScope Multiplex Fluorescent Assay kit (ACD Inc.) together with a specific probe complementary to a section of the mouse TH (NCBI Gene ID: 21823, ACD, 317621), PRR (NCBI Gene ID: 70495, ACD, 429931), and vasopressin (NCBI Gene ID: 11998, ACD, 401391) mRNA sequences. As a negative control, we used a probe for the bacterial gene 4-hydroxy-tetrahydrodipicolinate reductase (DapB) (NCBI Gene ID: EF191515, ACD, 310043). For RNAScope assays, WT C57Bl/6J mice (*n* = 3–6/group) were transcardially perfused first with 0.9% saline and then with 4% paraformaldehyde (PFA; Sigma-Aldrich). Thereafter, brains were immediately extracted and kept in 4% PFA for 24 hours at 4°C and then cryoprotected in 30% sucrose solution for an additional 24 hours at 4°C. Brains were frozen at –20°C in Tissue Freezing Medium (EMS, 72592) for 12 hours before sectioning. PVN-containing coronal sections (30 μm thickness) were cut using a cryotome (Leica CM1950) at –24°C. Sections were floated in PBS 1× containing 0.1% sodium azide as conservative at 4°C until used. Sections were then mounted onto Colorfrost Plus slides (Thermo Fisher Scientific, 12-550-18) and dried at –20°C for 30 minutes. The standard RNAScope protocol for fixed frozen tissues (ACD User Manual 323100-USM) was followed with minor modifications. A HybEZ II Hybridization System (ACD Inc.) was used to control incubation temperature. Briefly, slides were baked at 60°C for 25 minutes, before being fixed in PFA for 15 minutes at 4°C, followed by dehydration with a graded series of ethanol (50%, 70%, and 100%) for 5 minutes each at room temperature. A hydrophobic barrier was drawn surrounding the mounted tissue, and sections were incubated in 3% hydrogen peroxide for 10 minutes at room temperature and rinsed in distilled H_2_O. Target retrieval was performed by steaming slides (Hamilton Beach 37530A steamer) for 5 minutes. Slides were reimmersed in 100% ethanol for 3 minutes and air dried at room temperature. Sections were then incubated with Protease III for 30 minutes at 40°C. Excess liquid was removed from the slide, and sections were incubated with either a mixture of TH and PRR probes or negative control probe (DapB); slides were incubated at 40°C for 2 hours. For signal amplification, slides were incubated with AMP1 (30 minutes), AMP2 (30 minutes), and AMP3 (15 minutes) (ACD, 320851) at 40°C, with thorough washes in 1× wash buffer between steps. After amplification, a horseradish peroxidase–conjugated (HRP-conjugated) probe of interest was applied and incubated for 15 minutes at 40°C. This was followed by a 30-minute incubation at 40°C with fluorescein (1:250 dilution) for PRR and Cyanine 5 (1:250 dilution) for TH, followed by a 15-minute incubation with an HRP blocker at 40°C. Cell nuclei were counterstained with DAPI, after which sections were mounted and covered with glass slip using Prolong Gold Antifade Mountant (Thermo Fisher Scientific, P36930) and imaged using a confocal microscope (Leica Stellaris 8).

All images were acquired at a resolution of 1,024 × 1,024 with a pixel speed of 600 Hz. Pinhole, laser power, and gain were optimized and kept consistent for all slides, including negative control slides. For cell counting analysis, at least 4 images from each mouse with similar coronal levels covering rostral (bregma, –0.58 mm) to caudal (bregma, –1.22 mm) PVN were used for RNAScope as described above. Cells were considered to express the mRNA of interest if at least 3 visible transcripts, defined as an individual punctate dot, were observed surrounding a nucleus (58). Individual cells were identified using DAPI. Cell count and colocalization analysis were manually performed using Adobe Photoshop.

### Bilateral nanoinjection of AAVs into the PVN.

Mice were anesthetized using 4%–5% isoflurane in 100% O_2_ and flushed at 1 L/min for 2 minutes, and anesthesia was subsequently maintained using 1.5% isoflurane. The top of each mouse’s head was shaved and sterilized with alcohol wipes, after which the mouse was placed in a digital stereotaxic apparatus (Stoelting) and held in place by ear bars secured just above the ear canal. Heads of mice were then sterilized with alcohol wipes, and an incision (~2 cm) in the skin along the top of each head was made to expose the skull. The skull was cleaned with 3% hydrogen peroxide using cotton swabs, after which holes were drilled into the skull, and bilateral nanoinjections of viral constructs were performed at the stereotaxic coordinates, 0.6 mm posterior, ± 0.3 mm lateral, and 5.4 mm ventral to bregma. A 1 μL Neuros Hamilton syringe (32 gauge) attached to a UMP3 syringe pump (WPI) was fixed to the stereotaxic frame and used for AAV nanoinjections at a rate of 5 nL/s for 15 minutes for a total injection volume of 75 nL. After each injection to the PVN, the syringe needle was left in place for an additional 5 minutes before removing it to prevent backflow of the virus through the needle track. The wound was sutured, and postoperation analgesics (SR Buprenex, 0.6 mg/kg, s.c.) were administered. Mice were allowed 4 weeks to recover from surgery and to allow for the expression of AAV2.

For the PVN^TH^-PRRKO study, mice bearing both PRR-LoxP and tdTomato-LoxP alleles (PRR-tdTomato-LoxP mice) received a bilateral microinjection directed to the PVN of an AAV-expressing Cre-recombinase driven by a rat TH reporter (AAV2-rTH-Cre; 1.88 × 10^8^ vg in 75 nL, Vector Biolabs), while tdTomato-LoxP control mice lacking the floxed PRR allele underwent the same AAV2-rTH-Cre virus injection protocol and were used as controls (Figure 2A and Figure 3A). A complementary, second PVN^TH^-PRRKO study was performed in mice lacking the tdTomato reporter. Here, PRR^fl/fl^ mice received a bilateral microinjection to the PVN of either a control virus (AAV2-TH-GFP) or AAV2-rTH-Cre to delete PRR in PVN^TH^ neurons (Supplemental Figure 2A). For the chemogenetic study, hM4Di^fl/fl^ mice received bilateral stereotaxic injections to the PVN of either AAV2-TH-GFP (6.8 × 10^8^ vg in 136 nL, Vector Biolabs) as a control or AAV2-rTH-Cre (6.8 × 10^8^ vg in 272 nL, Vector Biolabs) to induce the expression of hM4Di in TH^+^ cells in the PVN. For mapping of PVN^TH^ neuronal projections, the GFP^fl/fl^ mice received a unilateral microinjection of AAV2-rTH-Cre (1.88 × 10^8^ vg in 75 nL, Vector Biolabs) directed to the PVN. Injection sites were confirmed at the end of each study by localizing fluorescence reporters. A mouse is defined as correctly targeted when the fluorescence reporter was observed in at least two-thirds of the PVN ranging from bregma –0.58 mm to –1.22 mm. Mice with either bilateral or unilateral targeting of PVN were included in the data analysis. In only a single mouse, viral microinjection failed to target PVN; this animal was excluded from further analysis.

### Fluorescent immunolabeling of PRR.

The PRR-tdTomato-LoxP mice and the tdTomato-LoxP mice used as control received a bilateral injection of AAV2-rTH-Cre virus as described above. Four weeks after the AAV2 injection, mice were transcardially perfused first with 0.9% saline and then with 4% PFA (Sigma-Aldrich). Thereafter, brains were extracted and kept in 4% PFA for 24 hours at 4°C and were then cryoprotected in 30% sucrose solution for an additional 24 hours at 4°C. Brains were frozen at –20°C in Tissue Freezing Medium (EMS, 72592) for 12 hours before sectioning. PVN-containing coronal sections (30 μm thickness) were cut using a cryotome (Leica CM1950) at –24°C. Sections were floated in PBS 1× containing 0.1% sodium azide as conservative at 4°C until used. Sections were blocked by incubation in 500 μL of PBST containing 10% normal goat serum for 1 hour at room temperature before incubation in primary anti-PRR antibody (NBP1-33605, Novus Biologicals) for 48 hours, diluted 1:100. Slices were then washed 3 times in PBS for 5 minutes each, followed by a 2-hour incubation with Alexa Fluor 488–conjugated goat anti-rabbit secondary antibody (A-11008, Invitrogen, Thermo Fisher Scientific), diluted 1:1,000 in PBST containing 2% normal goat serum. Slices were washed twice in PBS and incubated with DAPI (1:1,000) for 5 minutes. After 2 washes in PBS, slices were mounted on microscope slides and coverslipped using a fluorescent mounting medium (Vector Biosciences). Slides were light protected and allowed to dry overnight; they were imaged using a confocal microscope (Leica Stellaris 8). For data analysis, images from each mouse with similar coronal levels covering rostral (bregma, –0.58 mm) to caudal (bregma, –1.22 mm) PVN were used and analyzed by ImageJ (NIH; version: 2.1.0/1.53c) to identify the red tdTomato^+^ cells and the fluorescence intensity of the PRR immunolabeling in each tdTomato^+^ cell. The mean PRR fluorescence intensity of each tdTomato^+^ cell from 1 PVN was presented as 1 datum.

### Experimental model of DIO.

Mice were single-housed and fed a HFD (catalog D12492) or a matched LFD (catalog D12450J) from Research Diets Inc. containing either 60% or 10% Kcal from fat, respectively, for 6 weeks. Body weight and food intake were monitored weekly.

### FBG measurement.

FBG measurements were obtained from all mice at the end of the 6-week treatment with either HFD or a control diet. Mice were transferred to clean cages and fasted for 16 hours (5:00 p.m. to 9:00 a.m.). Mice were then restrained in a dark tube, and blood was collected directly onto a blood glucose strip from a tail vein following a small tail snip performed using a razor. Glucose was then measured using a Bayer 7393A Contour blood glucose meter (Bayer). Two measurements were performed for each mouse using blood from a tail snip, and the average of the 2 values was used as the final measurement.

### Glucose-tolerance test.

A glucose-tolerance test (GTT) was performed at the end of the 6-week treatment with either HFD or a control diet. Mice were fasted for 16 hours (5:00 p.m. to 9:00 a.m.). Baseline blood glucose (0 minutes) was measured, and mice were then injected i.p. with glucose (1 g/kg body weight) and administered as a 10% glucose in a 0.9% saline formulation. Blood glucose was measured 15, 30, 60, 90, and 120 minutes following the injection of glucose using a Bayer blood glucose meter, as described for FBG experiments.

### FSIGT.

At the University of Nevada, Reno, mice received a bilateral injection of AAV2-rTH-Cre into the PVN as described above. After 4 weeks of recovery, allowing for virus expression. Mice received 60% HFD for 6 weeks and were transported to the University of Washington at week 4 of HFD. The FSIGT was performed in collaboration with the NIDDK-funded Diabetes Research Center Metabolic and Cellular Phenotyping Core at the University of Washington, as previously described (38, 39). Briefly, mice underwent carotid artery and jugular vein catheterization and recovered for 7 days prior to FSIGT in the conscious state. For FSIGT, mice were fasted for 5 hours, and blood was sampled through the arterial catheter at t = –11 minutes and –1 minute, after which a bolus of 50% dextrose (1 g/kg) was delivered at t = 0 minutes through the jugular vein. Thereafter, blood was sampled through the arterial catheter at t = 1, 2, 3, 4, 5, 6, 8, 10, 12, 14, 16, 18, 20, 25, 30, 40, 50, and 60 minutes. Blood was measured using an Accu-Chek Aviva Plus glucometer (Roche). Plasma was collected for subsequent assay of insulin (Mouse Ultrasensitive Insulin ELISA, Alpco, 80-INSMSU-E01) and glucagon (Glucagon ELISA, Mercodia, 10-1281-01) levels. Experimental groups and treatment information were blinded to the Metabolic and Cellular Phenotyping Core staff. PVN targeting accuracy was not assessed in this cohort of animals, and no data were excluded from the analysis.

### Minimal model analysis of FSIGT data.

Plasma insulin and BG profiles during FSIGT were analyzed using MINMOD Millennium software to quantify insulin-independent glucose disposal, referred to as glucose effectiveness (S_G_) and S_I_, as previously described (38, 39, 59). The acute insulin response to glucose (AIR_G_) was calculated as the mean increment above basal insulin values measured between t = 0 and 4 minutes. The disposition index was calculated as the product of S_I_ and AIR_G_.

### Electrophysiological studies.

For PVN brain slice preparation, mice were anesthetized with ketamine (100 mg/kg; AmerisourceBergen) and xylazine (10 mg/kg; AmerisourceBergen) and sacrificed. Brains were quickly extracted from the skull and transferred into cold (4°C) aCSF containing (in mM): 124 NaCl, 1 MgSO_4_, 5 KCl, 1.25 KH_2_PO_4_, 10 glucose, 26 NaHCO_3_, and 2 CaCl_2_; bubbled with 95% O_2_/5% CO_2_ (pH 7.4). Coronal hypothalamic slices (250 μm thick) containing PVN were obtained with a VT1200 S microtome (Leica Microsystems) in cold, oxygenated aCSF. Coronal slices were then maintained for recovery for at least 45 minutes at room temperature (22°C–24°C) before the start of the electrophysiological recordings. For electrophysiological recordings, individual slices were transferred to a recording chamber (~0.5 mL of volume) and perfused with continuously bubbled (95% O_2_, 5% CO_2_) aCSF at a rate of 3 mL min^−1^. The flow rate was controlled by a Perfusion Valve Control System (Warner Instruments), and recording temperature was maintained at 30°C–32°C using a Dual Channel Temperature Controller (TC-344C, Warner Instruments). Voltage-clamp recordings were made with an Axopatch 200B amplifier with Digidata (Molecular Devices). TH^+^ cells in the PVN were visualized by epifluorescence (tdTomato Reporter) using a fixed-stage upright microscope Olympus BX54WI (Olympus Corporation) equipped with a water-immersion objective (60×; LUMPlan FL N, FN26.5, Olympus). Borosilicate glass (1.5 mm OD, 1.17 mm ID: BF150-117-10, P-87; Sutter Instruments) was used to prepare the recording electrodes of 2 to 4 MΩ with a horizontal puller (P-87; Sutter Instruments) and filled with aCSF solution. Assays were aborted if the series resistance was unstable throughout the recording (>20% change). Currents were low-pass filtered at 2 kHz and sampled at 10 kHz. An agar-KCl (1M) bridge was used to ground the recording chamber to the bath.

The cell-attached recordings were performed with extracellular aCSF solution. To isolate and record the SFR resulting from activation of voltage-gated cation channels, we abolished all the synaptic transmission by adding a GABA-A receptor antagonist (picrotoxin, 50 μM), an antagonist of AMPA/kainate receptors (CNQX, 5 μM), and an NMDA receptor antagonist (D-APV, 50 μM), which were obtained from Sigma-Aldrich. Stock solutions of the drugs were prepared and maintained at −20°C until use: picrotoxin (50 mM in ethanol), CNQX (5 mM in DMSO), and D-APV (50 mM in water). To obtain the final concentration, the stock solution was diluted in aCSF. All recordings were obtained from tdTomato^+^ cells from the TH^Cre+^tdTomato^fl/+^ PVN slices, using a combination of fluorescence illumination (excitation [Ex] = 554, emission [Em] = 581nm) and infrared differential interference contrast (IR-DIC) microscopy. The SFR was recorded in voltage-clamp mode with 0 mV of holding potential and a loose resistance cell (<1 GΩ). The effects of prorenin (PR, 2.5 nM) (ANASPEC) and PRO20 (250 nM, ChinaPeptides) on SFR were recorded. At the end of the experiments, tetrodotoxin (1 μM), a Na^+^ channel inhibitor, was added to the bath to abolish and confirm neural activity. Data are shown as mean ± SEM. The SFR was analyzed with Clampfit 11.1 (Molecular Devices).

### Continuous monitoring of blood pressure and heart rate by telemetry probes.

Two weeks after receiving the bilateral injection of AAV2-TH-Cre into the PVNs, the PRR-tdTomato-LoxP and the tdTomato-LoxP mice were implanted with telemetry radio transmitters (PA-C10, DSI, Harvard Bioscience Inc.) to continuously monitor blood pressure, heart rate, and locomotor activity levels in awake, freely moving mice (20, 30, 60). Briefly, mice were anesthetized using 4%–5% isoflurane in 100% O_2_ and flushed at 1 L/min for 2 minutes, and anesthesia was subsequently maintained using 1.5% isoflurane. The oblique and tracheal muscles were separated to expose the left carotid artery. The probe catheter was implanted into the left carotid artery and secured with a suture. The body of the radio transmitter was s.c. implanted in the right flank under the arm. Two weeks after recovery from surgery, blood pressure, heart rate, and locomotor activity were recorded continuously at baseline before HFD and following 6 weeks of HFD.

### Continuous glucose monitoring and DREADD activation of PVN^TH^ neurons.

One month following the bilateral injection of either AAV2-TH-GFP (control) or AAV2-TH-Cre in the PVN of hM4Di^fl/fl^ mice as described above, mice were implanted with radiotelemetry transmitters (HD-XG, DSI, Harvard Bioscience Inc.) to continuously monitor blood glucose levels in awake, freely moving mice. Briefly, mice were anesthetized using 4%–5% isoflurane in 100% O_2_ and flushed at 1 L/min for 2 minutes, and anesthesia was subsequently maintained using 1.5% isoflurane. The oblique and tracheal muscles were separated to expose the left carotid artery (19, 20, 31, 61). The glucose radio telemetry transmitter (HD-XG) catheter was implanted into the left carotid artery and secured with a suture. The body of the radio transmitter was s.c. implanted in the right flank under the arm.

Mice were allowed to recover from surgery for 1 week, and telemetry transmitters were calibrated twice a week according to the manufacturer’s recommendations. Briefly, the calibrations consisted of measuring blood glucose levels using a Nova StatStrip Xpress glucometer (Nova Biomedical) initially at baseline and following an i.p. injection of glucose (2 g/Kg). After that, baseline blood glucose was measured using a test strip twice weekly. This procedure collected blood directly on a blood glucose strip following a small tail snip performed with a razor. Blood glucose values were then entered into Ponemah software (DSI, Harvard Bioscience Inc.) and associated with current values at the specific time points in which blood glucose was measured. Following transmitter’s calibration, baseline blood glucose was continuously monitored, and mice were i.p. injected with either sterile 0.9% saline (vehicle) or Clozapine-N-Oxide Dihydrochloride (CNO, catalog 6329, Tocris), a water-soluble salt of CNO, at 1 mg/Kg at least 48 hours apart; blood glucose was continuously monitored.

### Statistics.

Data are expressed as mean ± SEM. Outliers were identified using robust regression and outlier removal (ROUT) method, coefficient *q* = 1% (FDR less than 1%), using GraphPad Prism Version 10 software. Data were analyzed by 2-tailed paired Student’s *t* test, unpaired Student’s *t* test, Mann-Whitney *U* test, or 2-way ANOVA with a mixed-effects model and Fisher’s least significant difference (LSD) tests to correct for multiple comparisons, as appropriate. Statistical comparisons were performed using GraphPad Prism Version 10 software. Differences with *P* < 0.05 were considered statistically significant.

### Study approval.

All procedures were conducted following the NIH *Guide for the Care and Use of Laboratory Animals* (National Academies Press, 2011) and were approved by the IACUC and the Institutional Biosafety Committee at the University of Nevada, Reno. The use of AAV was approved by the Institutional Biosafety Committee at the University of Nevada, Reno.

### Data availability.

Values for all data points in graphs are reported in the Supporting Data Values file.

## Author contributions

SP, LACS, CJW, MERM, and AJBG were involved in the experimental design, experimental procedures, data acquisition, data analysis, and manuscript writing and editing; they all contributed equally to the data collection and presentation, and thus, they are the co–first authors of the manuscript; the order of co–first authors was determined by the significance of their contributions ASS contributed to the electrophysiological experimental design. SGC was involved in RNAScope experimental technical support. DS was involved in data analysis and discussion of the results. RNB, GJM, MWS, and YFE participated in designing experiments, discussing results, and editing the manuscript. YFE is the guarantor of this work and, as such, has full access to all the data in the study and takes responsibility for the integrity of the data and the accuracy of the data analysis.

## Supplementary Material

Supplemental data

Supporting data values

## Version 1 02/13/2024

In-Press Preview

## Version 2 03/22/2024

Electronic publication
