# Supplementary material for: (Pro)renin receptor signaling in hypothalamic tyrosine hydroxylase neurons is required for obesity-associated glucose metabolic impairment
Source: JCI Insight. 2024 Feb 13;9(6):e174294. doi: 10.1172/jci.insight.174294 (PMC11063935; doi:10.1172/jci.insight.174294)
Supplement: Supplemental data [file jciinsight-9-174294-s189.pdf]

(Pro)renin Receptor Signaling in Hypothalamic Tyrosine Hydroxylase Neurons is Required for Obesity-Associated Glucose Metabolic Impairment

Shiyue Pan[1,2*], Lucas A.C. Souza[1,2*], Caleb J. Worker[1,2*], Miriam E. Reyes Mendez[1,2*], Ariana Julia B. Gayban[1,2*], Silvana G. Cooper[1,2], Alfredo Sanchez Solano[1,2], Richard N. Bergman[3], Darko Stefanovski[4], Gregory J. Morton[5], Michael W. Schwartz[5], and Yumei Feng Earley[1,2#]

1. Departments of Pharmacology and Physiology & Cell Biology, University of Nevada, Reno, School of Medicine, Reno, NV, USA.
2. Center for Molecular and Cellular Signaling in the Cardiovascular System, University of Nevada, Reno, Reno, NV, USA.
3. Diabetes and Obesity Research Institute, Cedars-Sinai Medical Center, Los Angeles, CA, USA
4. New Bolton Center, School of Veterinary Medicine, University of Pennsylvania Philadelphia, PA, USA.
5. University of Washington Medicine Diabetes Institute, University of Washington, Seattle, WA, USA.

*Contribute equally to the work

[#]Correspondence to:

Yumei Feng Earley MD, PhD, FAHA

Professor of Pharmacology and Physiology & Cell Biology

Center for Molecular and Cellular Signaling in the Cardiovascular System

University of Nevada, Reno, School of Medicine

1664 North Virginia Street, Mail-stop 0318

Reno, NV, 89557

E-mail: yumeifeng@med.unr.edu

Supplemental Figure S1.

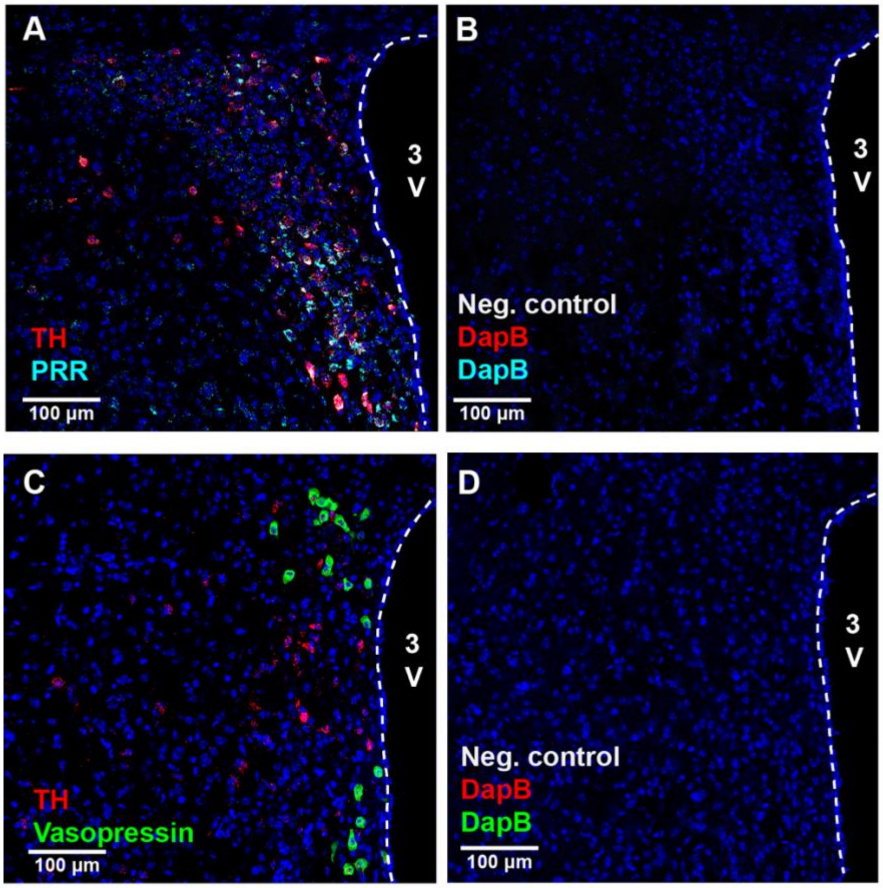

Figure S1. Validation of RNAScope *in situ* hybridization approach using bacterial gene DapB as negative control. (**A**) Representative images of RNAscope *in situ* hybridization of TH (channel 2, red), PRR (channel 1, cyan), and DAPI (blue) in the mouse PVN (Bregma -0.70 mm). (**B**) Representative images of RNAscope *in situ* hybridization of DapB (channel 2, red), DapB (channel 1, cyan), and DAPI (blue) in the mouse PVN (Bregma -0.70 mm). (**C**) Representative images of RNAscope *in situ* hybridization of TH (channel 2, red), vasopressin (channel 3, green), and DAPI (blue) in the mouse PVN (Bregma -0.82 mm). (**D**) Representative images of RNAscope *in situ* hybridization of DapB (channel 2, red), DapB (channel 3, green), and DAPI (blue) in the mouse PVN (Bregma -0.82 mm).

Supplemental Figure S2.

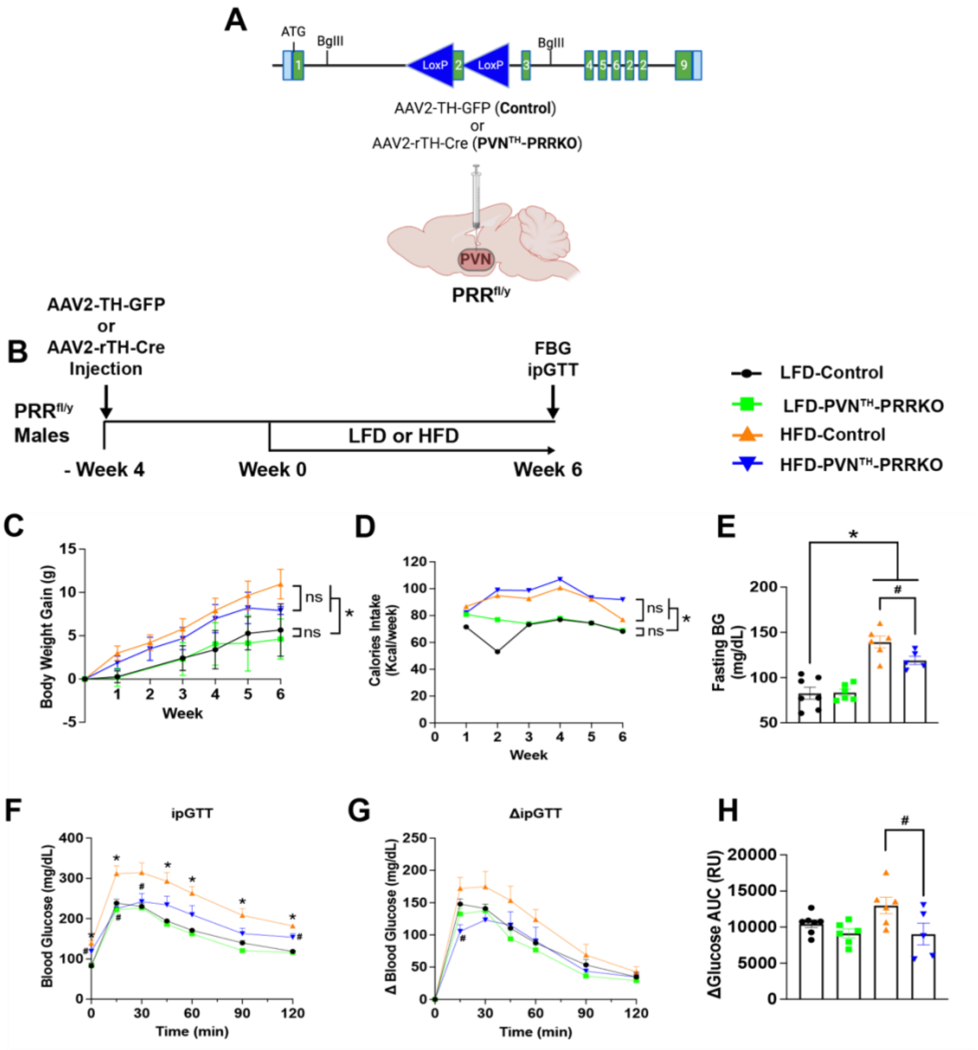

Figure S2. Deletion of (pro)renin receptor (PRR) in PVNTH neurons lowers fasting blood glucose (BG) and improves glucose tolerance in HFD-induced obese male mice without Tdtomato reporter. (**A**) Schematic representation of Cre-LoxP-mediated deletion of PRR in PVNTH neurons in male PRR$^{fl/y}$ mice. Adult male PRR$^{fl/y}$ mice received bilateral microinjection into the PVN of an AAV2-TH-Cre to delete PRR in PVNTH neurons (PVNTH-PRRKO) or control virus (AAV2-TH-GFP) as Control. (**B**) Schematic diagram of the experimental protocol. Four weeks after viral injection, mice were placed on either an LFD (10% calories from fat) or an HFD (60% calories from fat. (**C**) Weekly body weight gain, (**D**) calorie intake, (**E**) fasting blood glucose at 6 weeks following exposure to either an HFD relative to LFD, (**F**) Blood glucose levels, (**G**) changes in glucose levels and (**H**) area under the glucose curve (AUC) from baseline after i.p. injection of glucose. n = 4-7 animals/group; Data are expressed as mean ± SEM. **p* < 0.05 vs. LFD-Control, $^{\#}p$ < 0.05 vs. HFD-Control. 2-way ANOVA with Fisher's LSD multiple comparisons tests were used.

Supplemental Figure S3.

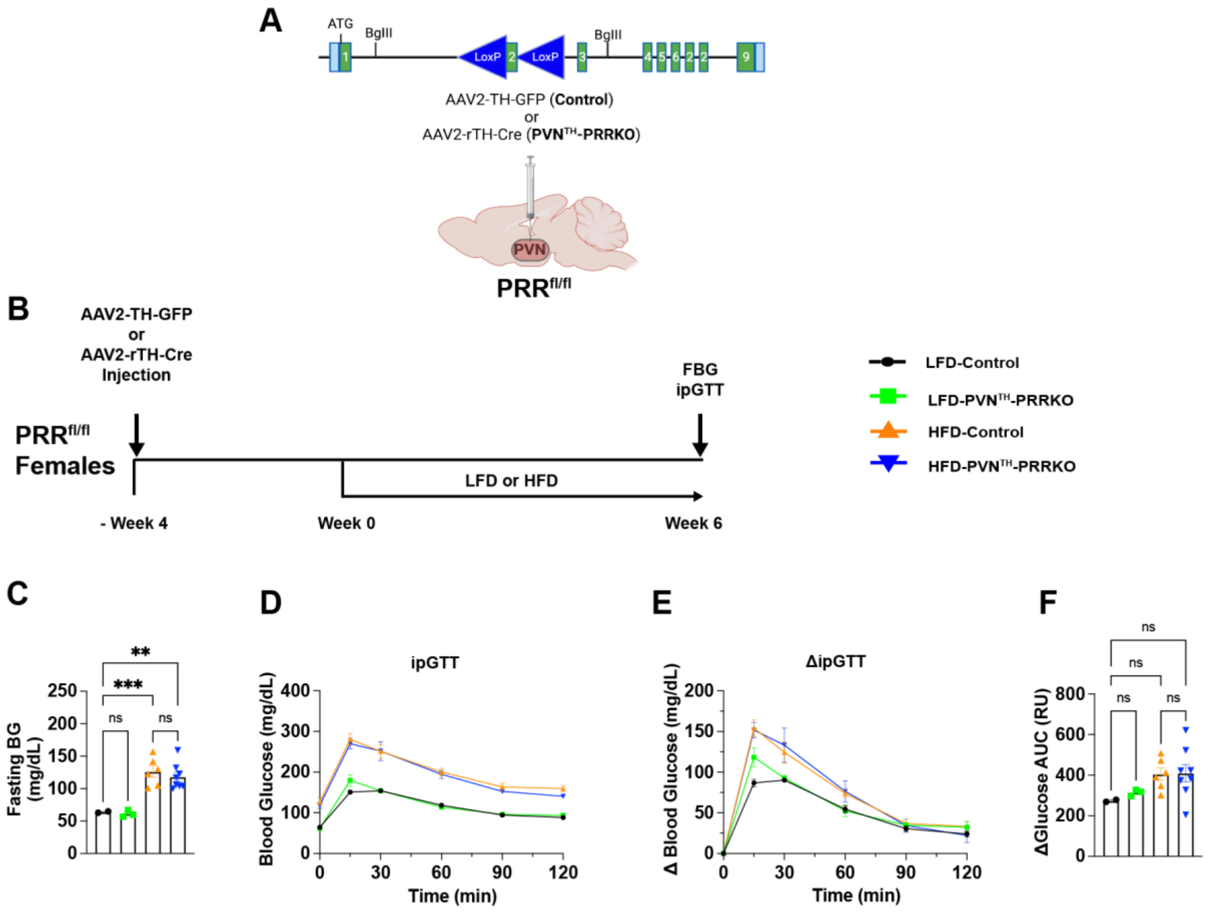

Figure S3. Effects of (pro)renin receptor (PRR) deletion in PVNTH neurons on fasting blood glucose (BG) and glucose tolerance in female PRR$^{fl/fl}$ mice. (**A**) Schematic representation of Cre-LoxP-mediated deletion of PRR in PVNTH neurons in female PRR$^{fl/fl}$ mice. Adult female PRR$^{fl/fl}$ mice received a bilateral microinjection into the PVN of an AAV2-TH-Cre to delete PRR in PVNTH neurons (PVNTH-PRRKO) or control virus (AAV2-TH-GFP) as Control. (**B**) Schematic diagram of the experimental protocol. Four weeks after viral injection, mice were placed on either an LFD (10% calories from fat) or an HFD (60% calories from fat). (**C**) Fasting BG at the end of 6 weeks, either HFD or LFD. (**D**) Blood glucose levels, (**E**) changes in glucose levels, and (**F**) area under the glucose curve (AUC) from baseline after i.p. injection of glucose. Data are expressed as mean ± SEM. ***p* < 0.01, and ****p* < 0.001. Lean-Control, 2-way ANOVA with Fisher's LSD multiple comparisons tests was used.

Supplemental Figure S4.

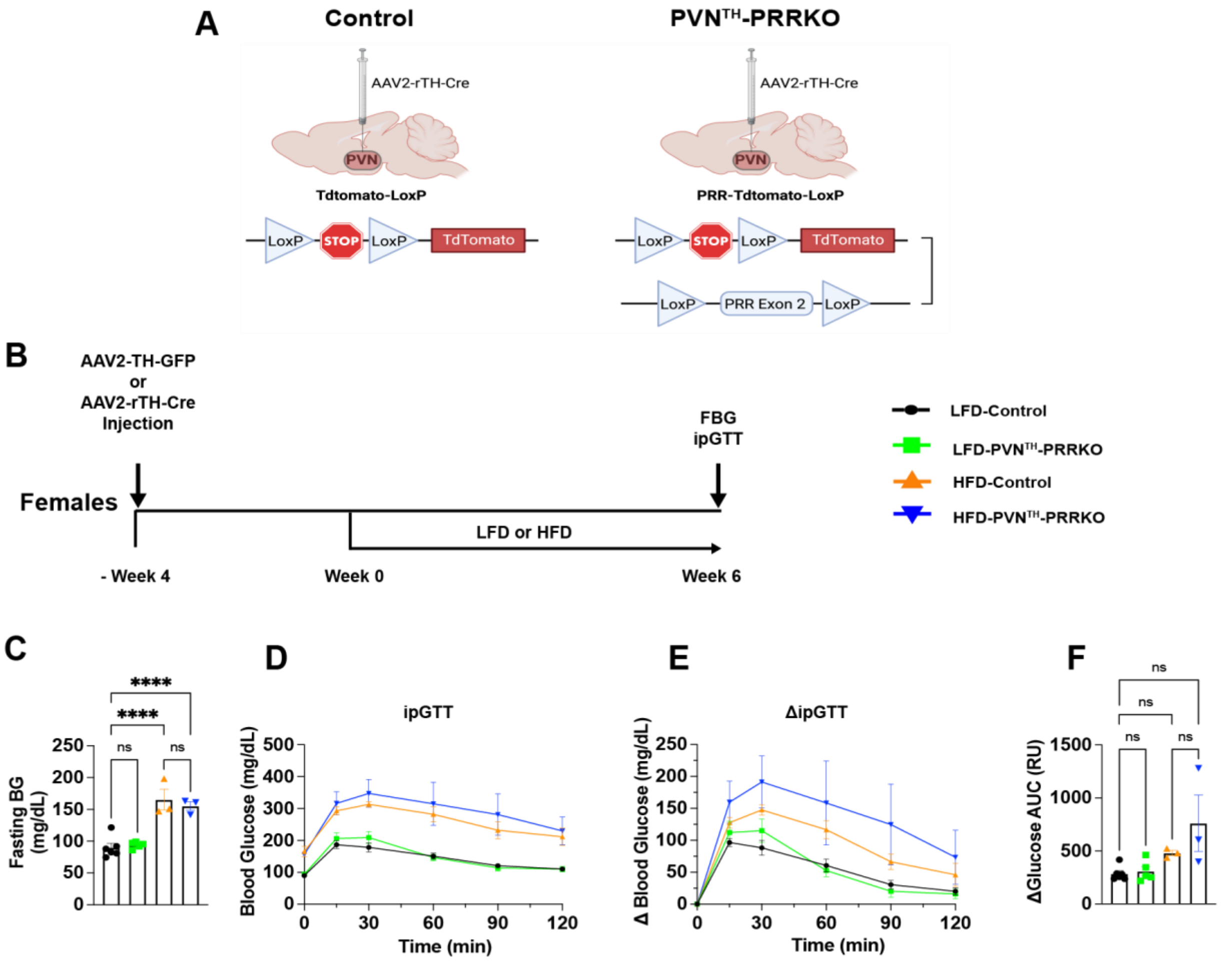

Figure S4. Effects of (pro)renin receptor (PRR) deletion in PVNTH neurons on fasting blood glucose (BG) and glucose tolerance in female PRR-Tdtomato-LoxP mice. (**A**) Schematic representation of Cre-LoxP-mediated deletion of PRR in PVNTH neurons in PRR-Tdtomato-LoxP mice. Adeno-associated virus expressing Cre recombinase driven by a rat tyrosine hydroxylase promoter (AAV2-rTH-Cre) was used to delete PRR and induce Tdtomato reporter expression in mice bearing both PRR-LoxP and Tdtomato-LoxP alleles showed as PVNTH-PRRKO. The Tdtomato-loxP mice that received the AAV2-rTH-Cre were used as Controls. (**B**) Schematic diagram of the experimental protocol. Four weeks after viral injection, mice were placed on either a low-fat diet (LFD, 10% calories from fat) or a high-fat diet (HFD, 60% calories from fat). (**C**) Fasting BG at the end of 6 weeks, either HFD or LFD. (**D**) Blood glucose levels, (**E**) changes in glucose levels, and (**F**) area under the glucose curve (AUC) from baseline after i.p. injection of glucose. Data are expressed as mean ± SEM. ****$p < 0.001$. Lean-Control, 2-way ANOVA with Fisher's LSD multiple comparisons tests was used.

Supplemental Figure S5.

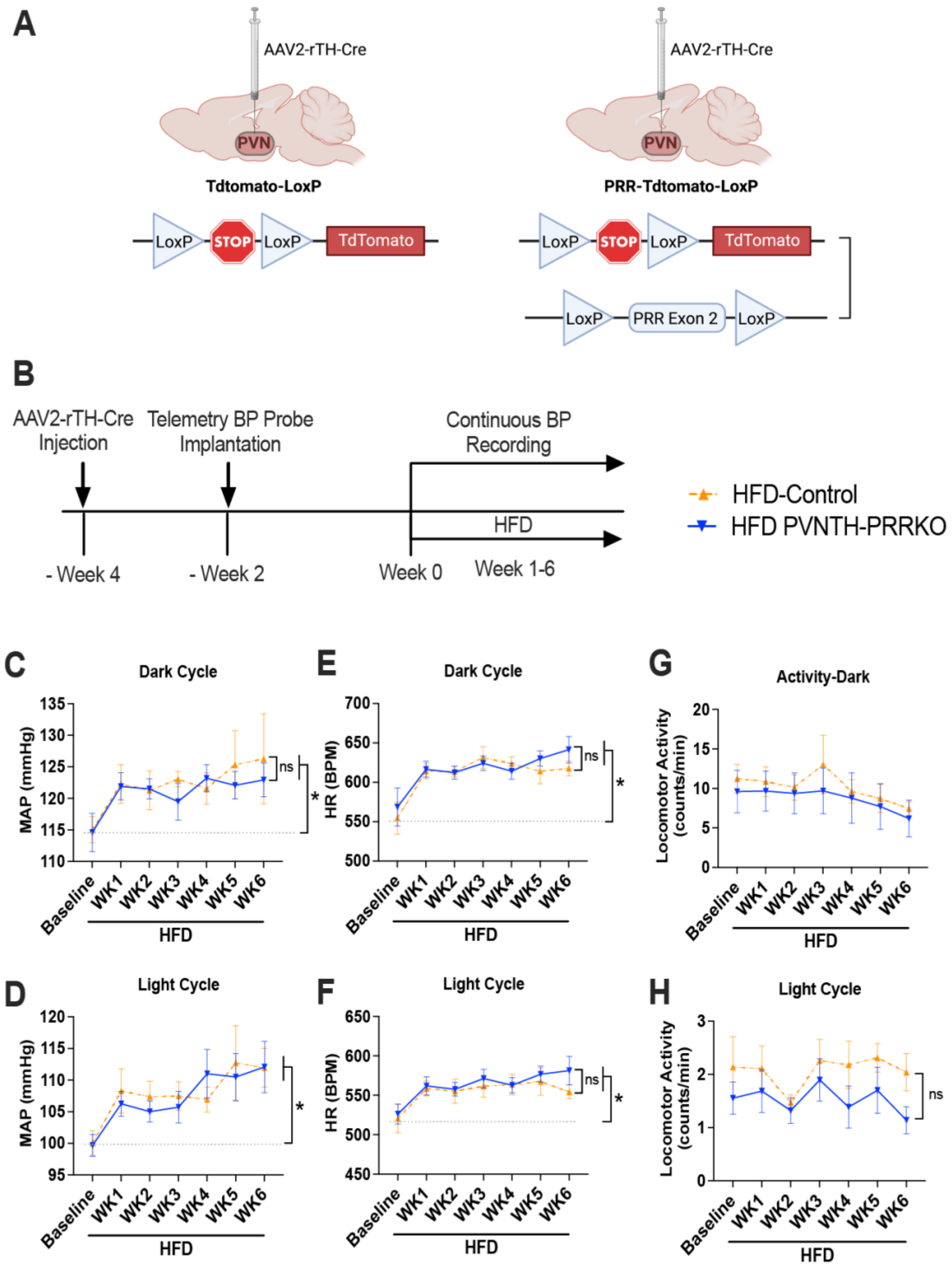

Figure S5. The effect of (pro)renin receptor (PRR) deletion in PVNTH neurons on blood pressure, heart rate, and locomotor activity in freely moving male mice recording by telemetry. (**A**) Schematic representation of Cre-LoxP-mediated deletion of PRR in PVNTH neurons in PRR-Tdtomato-LoxP mice. Adeno-associated virus expressing Cre recombinase driven by a rat tyrosine hydroxylase promoter (AAV2-rTH-Cre) was used to delete PRR and induce Tdtomato reporter expression in mice bearing both PRR-LoxP and Tdtomato-LoxP alleles showed as PVNTH-PRRKO. The Tdtomato-loxP mice that received the AAV2-rTH-Cre were used as a Control; (**B**) Schematic diagram of the experimental protocol. Mice were implanted with telemetric probes (model: PA-C10). Baseline parameters were recorded before the HFD regiment. Mice received HFD (60% calories from fat). (**C** and **D**) Mean arterial blood pressure (MAP) in dark (C) and light cycle (D). (**E** and **F**) Heart rate (HR) in dark (E) and light cycle (F). (**G** and **H**) Locomotor activity in the dark (G) and light cycle (H). Data are expressed as mean ± SEM, n = 5-6 mice/group. *$p < 0.05$ compared to the baseline in the same genotype, Two-way ANOVA with Fisher's LSD multiple comparisons tests was used.
